# A Case of Spontaneously Developed Retroperitoneal Parasitic Leiomyoma

**DOI:** 10.7759/cureus.71659

**Published:** 2024-10-16

**Authors:** Shunsuke Yasumi, Harunobu Matsumoto, Miho Sato, Eri Obata, Kaei Nasu

**Affiliations:** 1 Obstetrics and Gynecology, Nakatsu Municipal Hospital, Nakatsu, JPN

**Keywords:** laparoscopy, parasitic leiomyoma, peritoneal metaplasia, retroperitoneum, spontaneous development

## Abstract

Spontaneous retroperitoneal parasitic leiomyomas are extremely rare and are mostly located in the pelvic cavity. Herein, we present a case of a small primary parasitic leiomyoma arising in the retroperitoneum. A 46-year-old Japanese woman presented with iron deficiency anemia. The patient had not undergone any abdominal surgeries. Transvaginal ultrasonography and MRI revealed multiple uterine myomas and an 18-mm, low-intensity mass adjacent to the uterus. The patient underwent a total laparoscopic hysterectomy. A solitary retroperitoneal tumor in the pouch of Douglas was resected. Histologic examination of the resected retroperitoneal tumor revealed a well-circumscribed benign leiomyoma. In this case, we suggest that retroperitoneal primary parasitic leiomyoma may have arisen independently of uterine leiomyoma. Further investigations of similar cases may fully elucidate the pathogenesis of primary retroperitoneal parasitic leiomyomas.

## Introduction

Uterine leiomyoma is the most common benign gynecological tumor, affecting 20-50% of women of reproductive age and is the leading cause of hysterectomy [1, 2]. These benign neoplasms comprise smooth muscle cells and surrounding fibrous connective tissues. In addition to traditional patterns of leiomyomatous growth in the uterus, some unusual extrauterine growth presentations have been reported in the literature [3].

Parasitic leiomyoma is defined as the ectopic implantation of a uterine leiomyoma that loses its connection with the uterus. It usually arises in the pelvic or intra-abdominal organs, including the pelvic peritoneum, abdominal wall, small intestine, colon, and omentum, receives an alternative blood supply from another source, and grows afterward [3-7]. Parasitic leiomyomas can be divided into primary, spontaneous, secondary, and iatrogenic types. Primary parasitic leiomyomas are conventionally seen as a rare subtype of pedunculated subserosal fibroids that develop into large stalks. They adhere to surrounding structures, such as the broad ligament or omentum, and develop an auxiliary blood supply. Thus, leiomyomas can grow after detaching from the uterus [3-5]. Alternatively, primary parasitic leiomyomas are known to develop de novo with metaplasia of the peritoneum [3].

Here, we report a case of a small primary parasitic leiomyoma arising in the retroperitoneum and discuss the pathogenesis of the spontaneous development of this rare tumor.

## Case presentation

**Figure 1:**
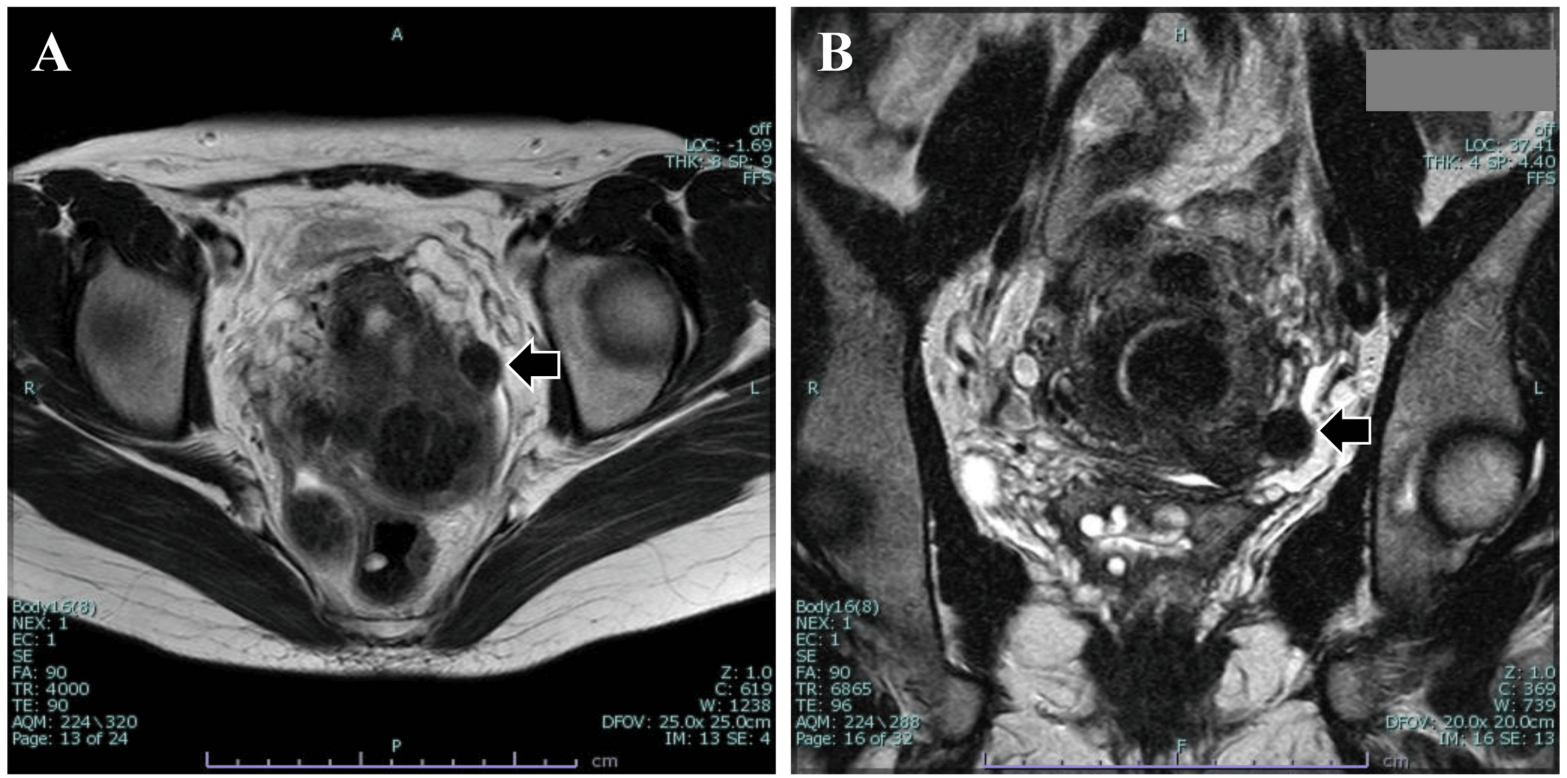
T2-weighted MRI images. (A) Horizontal view. (B) Coronal view. The MRI illustrates an 18-mm low-intensity mass adjacent to the uterus (arrow). However, the continuity of the uterus is unclear.

A 46-year-old Japanese woman (gravida 2, para 2) was referred to our hospital for a thorough examination of iron deficiency anemia. The patient had not undergone any abdominal surgeries. Transvaginal ultrasonography and MRI revealed multiple uterine myomas in subserous, intramural, and submucosal locations. Additionally, an 18-mm low-intensity mass adjacent to the uterus was detected (Figure 1). However, the continuity with the uterus remained unclear.

**Figure 2:**
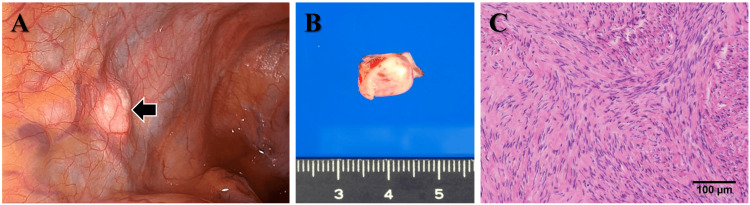
Laparoscopic, macroscopic, and histologic findings of the retroperitoneal tumor. (A) Laparoscopy reveals a retroperitoneal solid tumor on the left side of the pouch of Douglas (arrow). (B) The resected tumor is a white, well-circumscribed solid mass. (C) The resected tumor consists of whorled fascicles of benign smooth muscle cells separated by dense fibrovascular stroma.

After three months of treatment with a gonadotropin-releasing hormone antagonist, the patient underwent a laparoscopic total hysterectomy. During surgery, a solitary retroperitoneal tumor was found on the left side of the pouch of Douglas (Figure 2A). There was no continuity between the retroperitoneal tumor and the uterus. A few subserous and intramural leiomyomas were observed in the uterine corpus. Both ovaries and fallopian tubes were normal. Histologic examination of the resected retroperitoneal tumor revealed a well-circumscribed benign leiomyoma (Figure 2B-2C).

The patient’s postsurgical course was unremarkable, and she showed no evidence of recurrence nine months after the laparoscopic surgery. Written informed consent was obtained from the patient.

## Discussion

Lete I et al. [7] reviewed 274 patients with parasitic leiomyomas and found that 56% had no history of uterine surgery, whereas 44% had a history of hysterectomy or myomectomy. They estimated the prevalence of spontaneous and iatrogenic parasitic myomas to be 0.21% and 0.07%, respectively. The incidence of retroperitoneal parasitic leiomyoma is low. A PubMed search up to September 2011 revealed only 50 cases of retroperitoneal parasitic leiomyomas reported up to October 2008 [8-12]. Reportedly, 73% of retroperitoneal parasitic leiomyomas are located in the pelvic cavity [13].

These retroperitoneal parasitic leiomyomas may grow asymptomatically and can be diagnosed incidentally [14]. The most common symptom of a retroperitoneal parasitic leiomyoma is the palpation of a pelvic mass, which is present in almost 90% of patients [13]. Most published cases were large in size and surgically resected, with a high suspicion of malignancy or a misdiagnosis of subserous leiomyoma [15-18]. Most cases of small parasitic leiomyomas are found incidentally during other investigations or surgical procedures [19].

The pathogenesis of primary retroperitoneal parasitic leiomyoma remains controversial. The most accepted hypothesis involves the detachment of a pedunculated subserosal leiomyoma from the uterus. Alternatively, metaplasia of peritoneal mesothelial cells, retroperitoneal mesenchymal stem cells from embryonal remnants, and immature myocytes from the local vessel musculature are also considered potential sources for the development of de novo primary retroperitoneal parasitic leiomyomas [3, 17, 20]. These concepts appear to be suitable explanations for the present case because the leiomyoma was too small to have detached from the uterus.

Retroperitoneal parasitic leiomyomas are estrogen-dependent, slow-growing tumors with a low recurrence rate and are commonly reported in premenopausal women. We recommend that patients be followed up at least until menopause.

## Conclusions

We report a case of a small primary parasitic leiomyoma arising in the retroperitoneum. It has been suggested that some retroperitoneal primary parasitic leiomyomas may arise independently of uterine leiomyomas. Further investigations of similar cases may fully elucidate the pathogenesis of primary retroperitoneal parasitic leiomyomas.
